# Vascular basis of mucosal color

**DOI:** 10.1186/1746-160X-1-4

**Published:** 2005-08-24

**Authors:** Johannes Kleinheinz, André Büchter, Thomas Fillies, Ulrich Joos

**Affiliations:** 1 Department of Cranio-Maxillofacial Surgery, University of Muenster, Waldeyerstr. 30, D-48149 Muenster, Germany

## Abstract

**Background:**

Besides the color of the teeth the color of the alveolar gingiva plays a crucial role in esthetic rehabilitation in dento-alveolar treatment. Whereas nowadays the color of the teeth can be determined exactly and individually, the specific influence of the red color of the gingiva on treatment has not been assessed yet. The aim of this study was to evaluate the vascularization as the basis for gingival esthetics.

**Methods:**

Standardized photographs of defined areas of the alveolar gingiva in operated and non-operated patients were taken and assigned to groups with same characteristics after color comparisons. In addition, histologic and immunohistologic analyses of gingival specimens were performed for qualitative and quantitative assessment of vessels and vascularization. Finally, colors and number of vessels were correlated.

**Results:**

Our results demonstrated three different constellations of colors of the alveolar gingiva in healthy patients. The operated patients could not be grouped because of disparate depiction. There was a clear correlation between color and vessel number in the alveolar gingiva.

**Conclusion:**

Our investigations revealed the connections between vascularization and gingival color. Recommendations for specific change or even selection of colors based on the results cannot be given, but the importance of vascularly based incision lines was demonstrated.

## Background

Esthetic rehabilitation in dento-alveolar surgery was focused solely on reconstruction of position, shape and color of teeth for a long time. Significant improvement was achieved when reconstruction of form and volume of the peri- and paradental or periimplant soft tissue was added to the protocol. Esthetic impression depends on a coordinated interaction of red and white colors of dental and gingival structures. Nowadays the dental color is chosen in a very differentiated and individual way allowing the patient him/herself to select the color of the teeth to be replaced according to the neighbouring or missing teeth. In contrast the red color of the gingiva originates from acrylics, composite resins, silicones or porcelain-based materials which lack the range of differentiation of the white color. In cases without reestablishment of the red color the present red color is taken over regardless of color changes due to surgery. The role of the red color of the soft tissue is still unclear because of lack of complete fundamental knowledge, making definition of a starting point for a specific treatment of color changes impossible. The aim of this study was to evaluate the different mucosal and gingival colors and to classify them according to defined criteria. In addition, the vascular basis was analysed using histologic sections from the oral mucosa and compared with the colors.

## Methods

### Clinical evaluation

**Figure 1:**
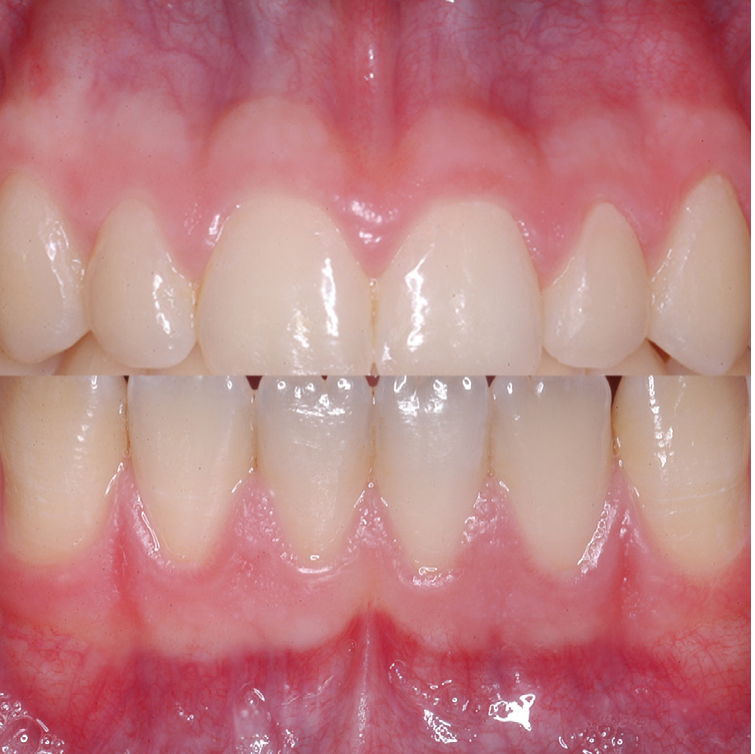
Demonstration of region of interest in the maxilla and mandible. There is a clear horizontal formation of different red coloring.

**Figure 2:**
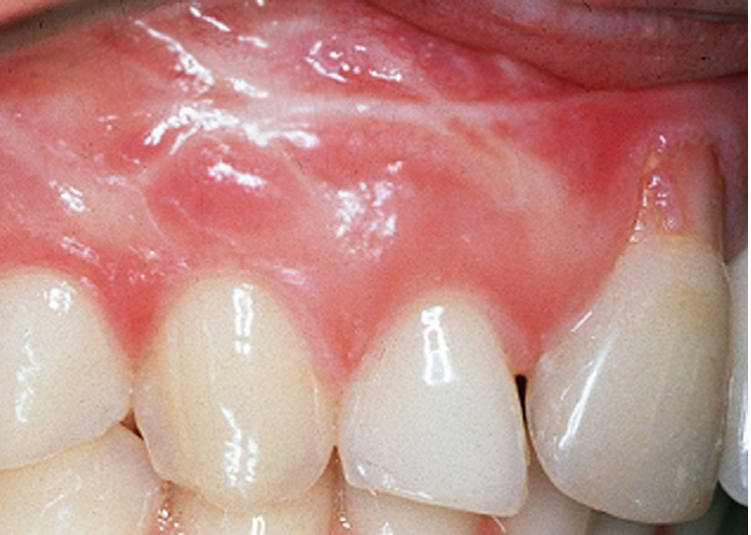
Paramarginal scars produce a complete different pattern of colors in comparison with healthy mucosa.

**Figure 3:**
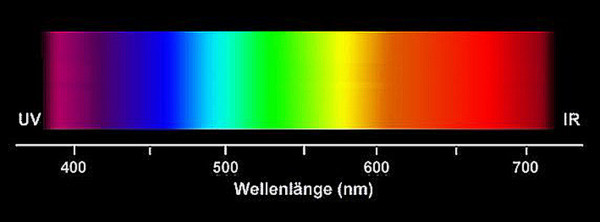
A standardized color scale was the basis for the determination of the wave length to compare the different coloring.

Standardized digital photographs of the maxillary and mandibular gingiva (#13 to 23 and #33 to 43) (Fig. 1) of healthy unoperated and healthy operated dentulous patients were taken (Minolta 3CCD digital with AF 50 macroobjective and ring flash). Healthy unoperated patients without previous surgery in the areas of interest were selected. Healthy operated patients had scars of the mucosa after surgery. The scar had to be in the defined areas, at least as long as two teeth and at least one year old (Fig. 2). None of the patients of both groups did show any sign of periodontal or gingival infection. The photographs were analyzed according to the following criteria: colors of attached and unattachedgingiva and color of the muco-gingival line(linea girlandiformis). Standardized color assignment was achieved by adjustment of the digital pictures using a standardized color scale and accordant wave length (Image Tool 3.0, University of Texas Health Center, San Antonio, USA) (Fig. 3). For each patient the color of the attached and unattached gingiva was determined, separately for maxilla and mandible.

### Histology and immunohistology

Specimens of the gingiva were taken during tumor surgery. Intraoperatively, the area of interest had been photodocumented before tumor resection. Gingival specimens were taken after the pathologist had evaluated the borders of the block and excluded any tumorinfiltration. Coloration was also classified using the same color scale as described above. After fixation and paraffin-embedding, specimens were cut demonstrating the whole area of the gingiva (attached and unattached). By means of immunohistological staining of the vessels using CD31 (DAKO, Hamburg, Germany) distribution of the vessels inside the gingiva was evaluated qualitatively in each specimen. The number of vessels was counted in 5 randomly selected so-called hot spots [1] for both keratinized and non-keratinized areas with defined magnification.

Analyses covered qualitative description of the distribution of the vessels in the different parts of the gingiva, quantitative assessment of the number of vessels in the keratinized and non-keratinized areas. Statistical analysis covered the comparison of distribution of vessels using t-test (significance level p < 0.05), and correlation between number of vessels and wave length/color of the different areas of the gingiva using a linear Spearman correlation analysis and calculation of coefficient of correlation.

## Results

Standardized photographs from 54 healthy unoperated patients and 32 operated patients were analysed. For histological and immunohistological analyses 28 gingival specimens were available.

### Clinical evaluation

In almost all patients characteristic formations of alveolar soft tissue was found which was divided into area of attached gingiva (gingiva propria), transition zone (muco-gingival line, linea girlandiformis) and unattached area (alveolar mucosa, gingival mucosa). This subdivision was not only because of mobility and surface structure but especially because of nuances of the red color. It was demonstrated that the muco-gingival line does not represent an independent anatomic structure with an own coloring but does appear as expression of the transition from attached to unattached gingiva, demonstrating the result of structural and color-coordinated changes.

### Qualitative evaluation and classification

**Figure 4:**
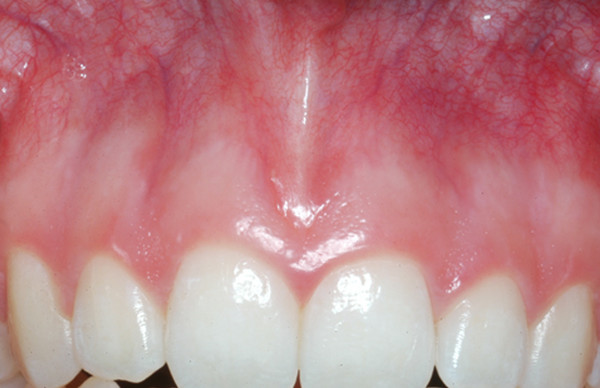
Group 1 showed a light presentation in both areas, the mucogingival line was not distinct.

**Figure 5:**
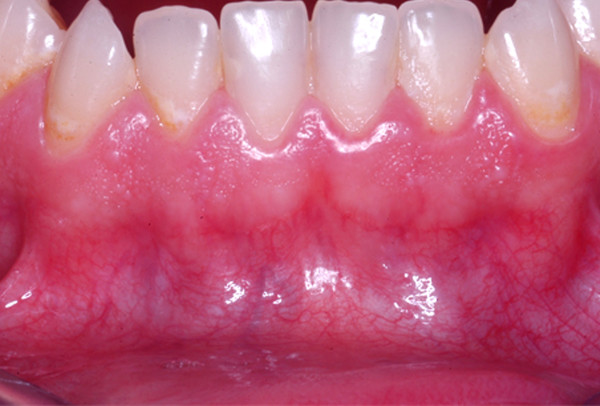
The second group was dominated by a dark color, the mucogingival line was also not pronounced.

**Figure 6:**
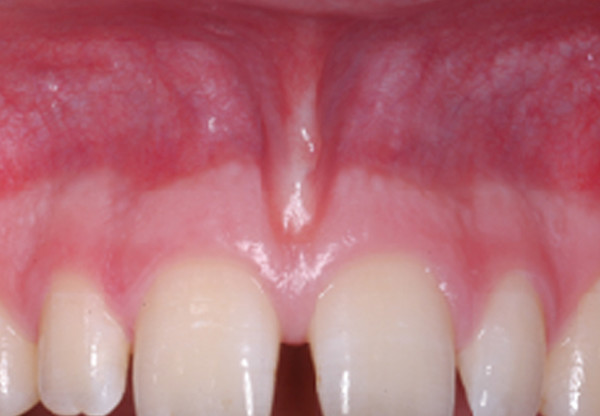
The distinct mucogingival line in the third group is the consequence of the marked changes of coloring.

**Figure 7:**
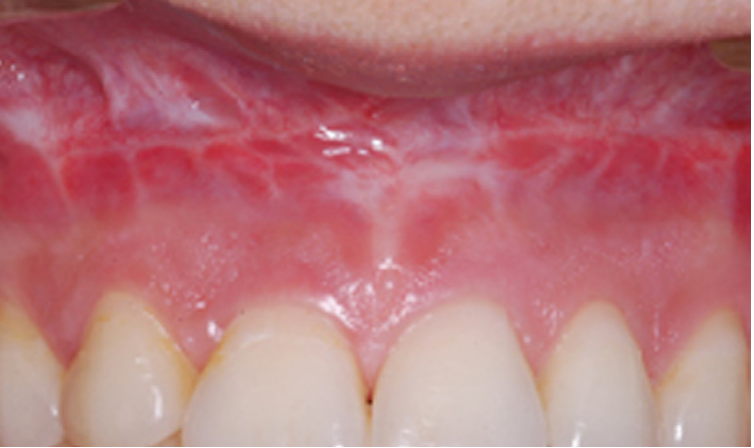
Scar tissue led to loss of horizontal formation of the red colors. Building up of a classification in operated cases was not possible.

Qualitative evaluation of the photodocumentation according to the mentioned criteria resulted in three subgroups of the healthy unoperated patient cohort. These subgroups were again subdivided according to the intensity of the muco-gingival line (Table 1). Group 1 generally demonstrated pale coloring of both gingival parts (Fig. 4), group 2 showed pronounced coloration of both regions (Fig. 5) and group 3 had a distinct color difference between both gingival parts (Fig. 6). With the dark-colored part always appearing in the area of the unattachedand the light-colored part in the area of the attached gingiva. The photographs of the operated patients (Fig. 7) did not allow any grouping or classification because of scar formation and thus strongly different colors. It was demonstrated that the naturally horizontally aligned layering of color nuances was strongly disturbed by scarring, leading to loss of defined transition zones.

**Table 1:** Distribution of patients on three clinical different groups. Mucogingival line was distinctive in cases of pronounced changes of coloring in group 3

	**mucogingival line**	
**mucosa**	distinct	not distinct

group 1: light	4	10
group 2: dark	5	12
group 3: combined	16	7

### Histological evaluation

**Figure 8:**
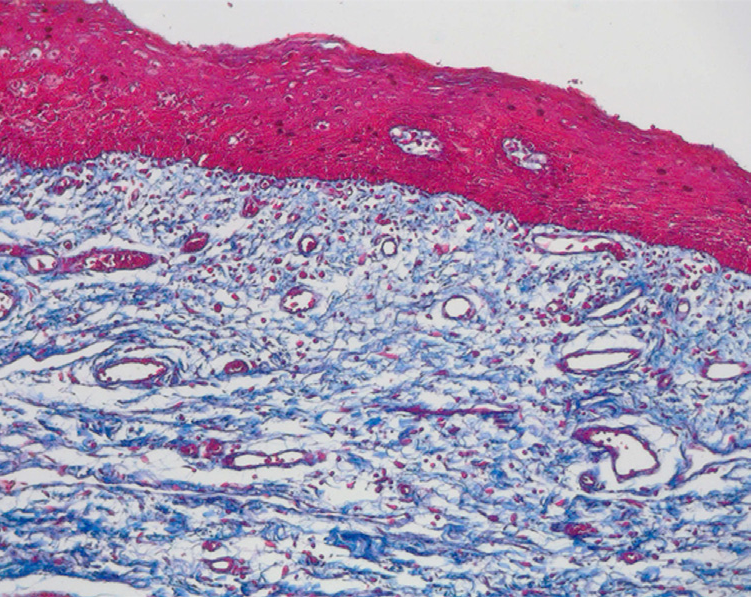
Histologic section (Azan staining, magnification ×10) demonstrates the vascular area in the submucous lamina propria.

The natural structure of the gingiva was evaluated histologically including keratinized and non-keratinized areas. The vessels were clearly demonstrated in both areas and their distribution analyzed. There was a strongly vascularized area under the epithelium layers in the lamina propria of the mucosa (Fig. 8).

### Quantification and correlation

**Figure 9:**
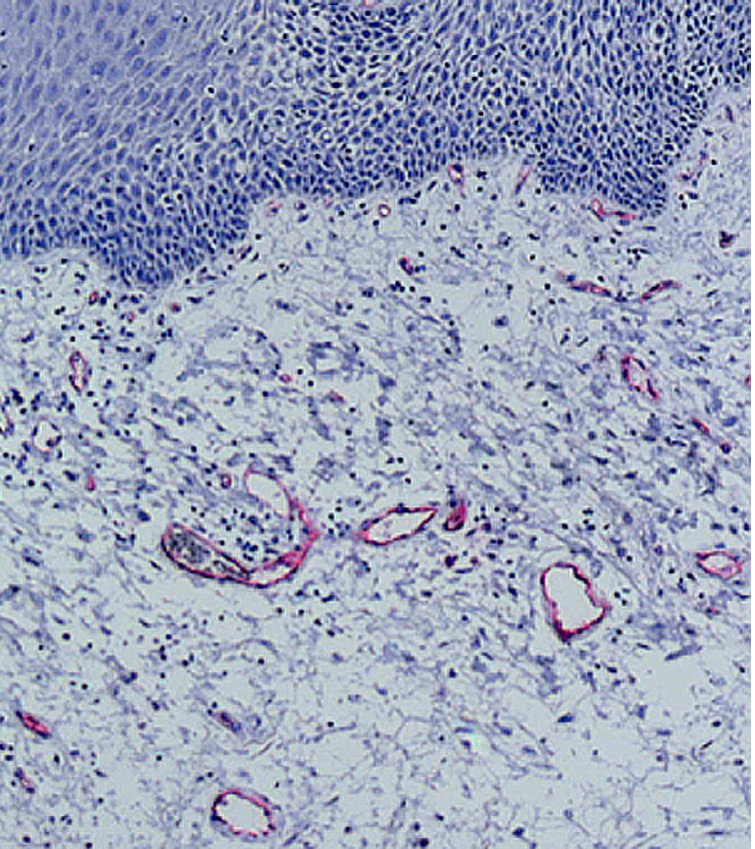
Immunohistologic staining of endothelial cells with CD 31 was the basis for evaluation of number of vessels.

**Figure 10:**
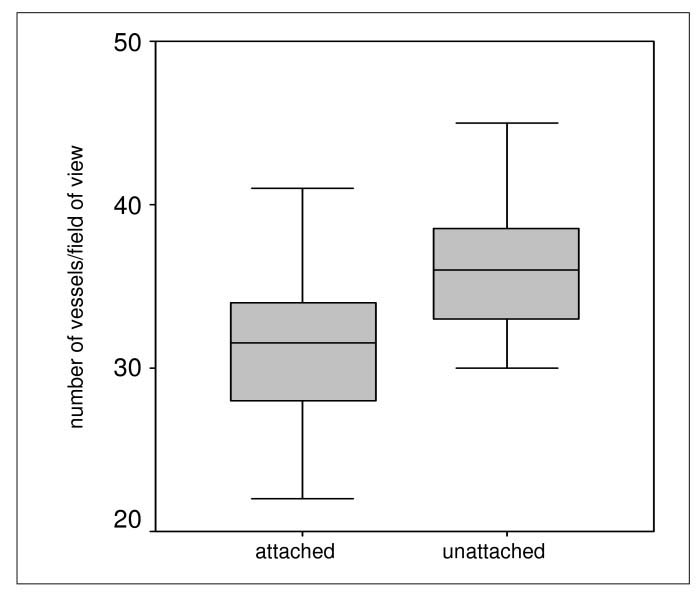
Numbers of vessels in attached and unattached areas of the gingiva showed no significant differences.

**Figure 11:**
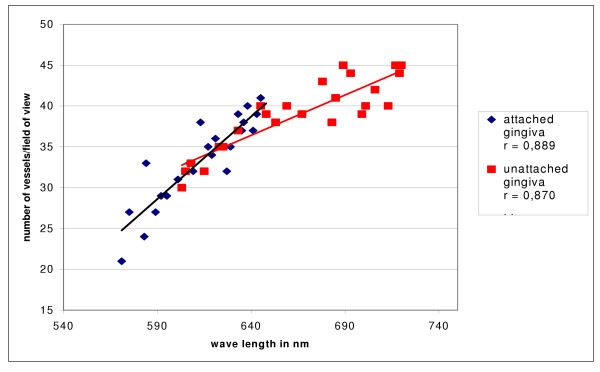
There is a distinctive positive correlation between number of vessels and color (defined as wave length) in both attached (coefficient of correlation r = 0,889) and unattached gingiva (coefficient of correlation r = 0,870). The higher the number of vessels the darker the color and the higher the wave length. Areas of attached gingiva showed more light colors on average.

Using immunohistological staining the endothelial cells were marked specifically and the vessels analyzed quantitatively (Fig. 9). The number of vessels showed slight differences concerning keratinization of the gingiva but these were not statistically significant (Fig. 10). The small number of vessels in the area of the attached and keratinized gingiva has to be seen in context with the overall thinner mucosal layer in the marginal area. In both groups small number of vessels resulted in significantly lighter, high number of vessels in significantly darker coloration. For each area, statistical analyses showed a significant correlation between number of vessels and wave length (Fig. 11).

## Discussion

To date scientific evaluation of the gingival mucosa concentrated on two fields both disregarding the color. On the one hand, vascularization of oral mucosa was investigated using different techniques (perfusion and plastination [2,3], histology [4], laserdoppler flowmetry [5], fluorangiography [6]), and, in particular, under the aspect of changes caused by local infections [7-9] or general diseases [10,11]. At this the evaluations were far beyond simple morphological descriptions and reached the level of molecular and genetical changes [11,12]. On the other hand, the muco-gingival or alveolar soft tissue surgery became more and more important especially in implantology aiming at optimal esthetic outcome. Main emphasis was placed on incision design, flap design and flap raising [13], soft tissue grafts [14-16] and microsurgical techniques. Targets were substitution of volume and structure and shape design but not the correction or change of color.

The combination of reconstruction/rehabilitation and coloration, which must arise from the causal interrelationship, was mentioned only to a small extent, still lacking direct therapeutical influence of the gingival color.

The natural changes of color from the attached to the unattached gingiva can be traced back to two factors: the different degrees of keratinization and the degree of vascularization. The more keratinization the attached gingiva shows the less gleaming of the vessels and the more ligth-colored the gingiva. Keratinization can strongly differ interindividually and mainly depends on mechanical loading and degree of elimination of dead cell layers.

The degree of vascularization also correlates to local mechanical loading and host response. Acute and florid periodontal and gingival infections lead to a hyperemia resulting in change of color [7]. The more distinct the infections, the darker the red color will be.

Considering the results of this study and those published in the literature it seems reasonable to avoid any changes of the red color of the gingiva during treatment, if possible, because currently there are no adequate tools or techniques to change the color in a way that is adapted to the individual situation and provides stability. In addition, it is necessary to optimize incisions in every single case to avoid pronounced changes of the natural anatomy and, thus local vascularization. That means that esthetically critical regions like the upper anterior area should not be incised obliquely or perpendicular to the course of the vessels. The incision must not disturb or interrupt the main vascularization inside the gingiva. Investigations by Cranin [17] clearly demonstrated that unfavorable incisions lead to disorientation of vessels and, therefore, delayed wound healing, scar formation and bone loss. Reorientation and regeneration of vessels will lead to changes of the color which will rarely adapt to the individual situation. Traumatisingpreparation techniques can end up in a subepithelial fibrosiswhich also influences the coloration. Microsurgical techniques and the use of appropriate instruments and magnification led to more reliable and stable color results.

Medicamentous therapies of periodontal diseases [18] or general diseases [19] were analyzed concerning their impact on morphological changes of the epithelium and the connective tissue and the degree of vascularization however recommendations for distinct treatment of colors could not be derived from these results.

## Conclusion

Conservation or rehabilitation of the red color of the gingiva can only be achieved by preserving the natural structures, precise and atraumatic interventions and avoidance or treatment of any inflammatory sources. The red coloration depends on the degree of keratinization and the distribution and the number of blood vessels. A distinct and durable influence on the color by surgical or medicamentous treatments seems to be impossible at present. It is recommended to change the soft tissue as less as possible during treatment to preserve natural and individual coloration.

## Competing interests

The author(s) declare that they have no competing interests.

## Authors' contributions

JK set up the design of the study, performed the surgical part, and helped to draft the manuscript. AB carried out the photographical analysis and performed the statistical analysis. TF carried out the histological and immunohistological studies and performed the statistical analysis. UJ participated in the design of the study, coordination of the patients and helped to draft the manuscript. All authors read and approved the final version of the manuscript. AB and TF contributed equally to this work.
